# Supplementary material for: Conditional chemoconnectomics (cCCTomics) as a strategy for efficient and conditional targeting of chemical transmission
Source: eLife. 2024 Apr 30;12:RP91927. doi: 10.7554/eLife.91927 (PMC11060718; doi:10.7554/eLife.91927)
Supplement: Figure 3—source data 3. [file elife-91927-fig3-data3.zip › Figure 3 source data3 part 2.zip!/Figure 3-Source data 3-part2/1025-13-CRISPResso_Report_ack040l3.zip!/CRISPResso_on_1025_13/4d.Position_dependent_average_indel_size.pdf]

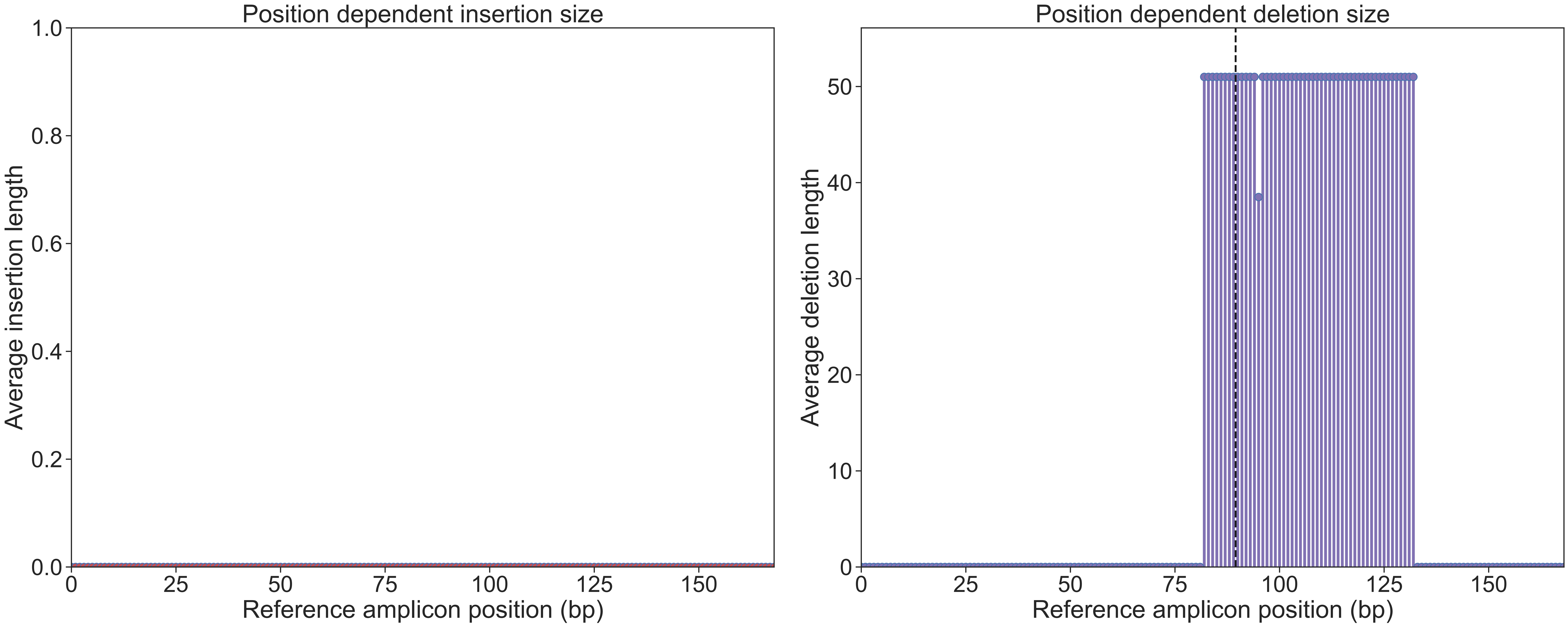
Position dependent insertion size
Average insertion length
0.0
0.2
0.4
0.6
0.8
1.0
0
25
50
75
100
125
150
Reference amplicon position (bp)
Position dependent deletion size
Average deletion length
0
10
20
30
40
50
0
25
50
75
100
125
150
Reference amplicon position (bp)
